# Odor-based context-dependent memory: influence of olfactory cues on declarative and nondeclarative memory indices

**DOI:** 10.1101/lm.053562.121

**Published:** 2022-05

**Authors:** Agnieszka Sorokowska, Marie Nord, Michał Mikołaj Stefańczyk, Maria Larsson

**Affiliations:** 1 Gösta Ekman Laboratory, Department of Psychology, Stockholm University, SE-106 91 Stockholm, Sweden; 2 Smell and Taste Research Lab, Institute of Psychology, University of Wroclaw, 50 597 Wroclaw, Poland

## Abstract

Reinstating the olfactory learning context can increase access to memory information, but it is not fully clear which memory functions are subject to an enhancing odor context reinstatement effect. Here, we tested whether congruent odor context during encoding and recall positively affected declarative and nondeclarative memory scores using a novel method for manipulation of an odorous environment; namely, intranasal Nosa plugs. Recall of a text and a complex figure as well as performance in a priming task were assessed immediately and 1 wk after encoding. We found that congruent odor exposure at encoding and recall aided free retrieval of a story at delayed testing but had no significant effect on a complex figure recall or a word completion task. Differences between the assessed memory indices suggest that olfactory environmental cues may be primarily efficient in free verbal recall tasks.

Our memories of events result from experiences that occurred within a complex physical and mental environment and, consequently, a memory representation consists of many different types of information (e.g., spatial and temporal) originating from several different senses. Relatedly, research in the area of context-dependent memory suggests that retrieval of specific episodes or information improves when the context present at encoding (learning) and retrieval is the same. Indeed, evidence suggests that access to memory information increases when the original learning context is reinstated (Smith and Vela 2001), and the encoding specificity principle additionally specifies that only the contextual cues available at encoding can aid retrieval (Tulving and Thomson 1973). Congruent factors at retrieval and encoding may act as memory cues—or “triggers”—for the original learning event that eventually may promote a better retrieval of the target information.

The study of context-dependent memory has gained empirical support from work that has used a range of different ambient (Bell et al. 1984; Mead and Ball 2007) and environmental (Smith and Vela 2001; for review, see Isarida and Isarida 2014) contexts. While evidence is rather scarce, most research suggests that also olfactory stimuli can serve as effective contextual cues, enhancing the recollection or recognition of previously encountered information (Willander and Larsson 2006, 2007; Arshamian et al. 2013; Larsson et al. 2014). A fascinating experiment by Aggleton and Waskett (1999) showed that reinstatement of unusual odors, forming a part of displays in a Viking museum, aided the recall of the content of these displays even after several years from the original visit in the museum. Given these particular, long-lasting effects of olfactory triggers and the general ability of odors to evoke emotionally loaded memories (Herz 1998; Toffolo et al. 2012) many existing studies focused on the area of autobiographical memories (see Chu and Downes 2000; for a review, see Hackländer et al. 2019). For example, odors were found to be particularly effective in evoking rich childhood memories (De Bruijn and Bender 2018). Smells may also aid socially relevant memory processes, with body odors aiding facial recognition (Cecchetto et al. 2020).

Odor-based context effects may also be of high interest in the learning context. Reinstated smells were shown to effectively enhance performance in a number of studies targeting declarative and direct measures of memory, including recall (Parker and Gellatly 1997; Lwin et al. 2010; Tamminen and Mebude 2019), recognition (Cann and Ross 1989; Parker et al. 2001; Cecchetto et al. 2020), procedural memory (Parker et al. 2001; Suss et al. 2012), and relearning performance (Smith et al. 1992). Odor effects were observed regardless of affective congruence with the encoded information (Hackländer and Bermeitinger 2017). Interestingly, olfactory cues were often found to be more effective triggers than other sensory stimuli, showing a particular value of odors as memory cues (but see Herz 1998). For example, odors helped retrieve details of an aversive film better than auditory, but not visual, stimuli (Toffolo et al. 2012), and prose recall performance was selectively higher following olfactory, but not visual, reinstatement (Pointer and Bond 1998). Lwin and collaborators (Lwin et al. 2010; Lwin and Morrin 2012) also showed that odors can not only serve as cues facilitating information recall, but also enhance the value of (concurrently presented) pictures as memory cues. Some studies have investigated whether reinstatement of olfactory context also affects nondeclarative or indirect measures of memory (i.e., when participants are unaware of the link between the learning phase and recall), and available evidence is mixed. Using a word stem completion paradigm, Schab (1990) found higher completion rates when chocolate and mothball odors were present at both encoding and recall 24 h later, and Ball et al. (2010) showed that word fragment completion was positively affected by a reinstatement of an unpleasant and distinctive odor.

However, the effects of odor reinstatement on memory are far from uniform. No facilitating effects of a congruent odor environment were observed in free recall and recognition of abstract figures (Reichert et al. 2017) or repeated solving of a previously encountered cognitive problem (Parker et al. 2001), nor was it found for accuracy of magic show reports in children (Roos af Hjelmsäter et al. 2015). Similar to the area of declarative memory studies, the findings on implicit memory are not consistent either. The significant effects reported by Ball et al. (2010) and Schab (1990) were not observed by Parker et al. (2000) and Ludvigson and Rottman (1989). Based on the existing literature, it is thus not entirely clear whether memory is indeed sensitive to the olfactory cues’ influence.

It should be noted here that there are certain problems that may contribute to the mixed evidence in the context of odor reinstatement effects. Researchers often addressed different cognitive domains in separate experiments rather than concurrently within the same study. Drawing general conclusions can be further hindered by differences between applied odors and their pleasantness (Schab 1990; Parker et al. 2000; Ball et al. 2010), as well as a relatively large variation in the mode of odor presentation and time elapsed between encoding and recall across. We decided to address these problems and assess odor reinstatement effects on various aspects of memory performance in a single study comprising three memory indices and a novel method of olfactory stimulation: odorized nose plugs (Nosa; https://nosaplugs.com). Young adults were presented with two episodic memory tests assessing recall of verbal (prose) and visuospatial (complex figure) information, and we also explored nondeclarative memory performance by means of a priming test with a word fragment completion task (Tulving and Schacter 1990; Karlsson et al. 2003). We wanted to examine whether a uniform reinstated odorous contextual cue, applied in standardized conditions in a paradigm involving several memory tests, will enhance performance in all tested memory domains. We were particularly interested in long-lasting effects of olfactory triggers, and therefore the memory performance was assessed twice: immediately after encoding and 1 wk later.

## Results

Descriptive statistics for each analyzed memory domain in all experimental groups are presented in Table 1.

**Table 1.**
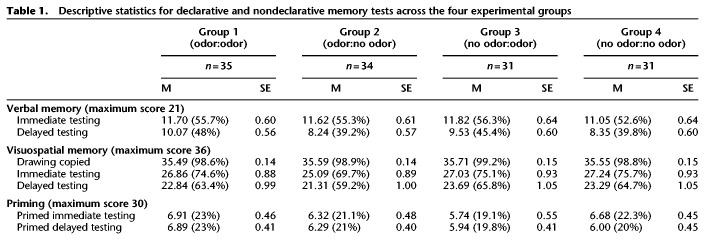
Descriptive statistics for declarative and nondeclarative memory tests across the four experimental groups

### Verbal episodic memory

There was no main effect of group on the performance in the RBMT episodic memory test, but we found a significant main effect of the testing session [*F*_(1,126)_ = 26.43, *P* < 0.001, η^2^_p_ = 0.173], with participants in all groups recalling significantly more information during the immediate testing session (see Table 1). We also observed a significant interaction between group and testing session of a small to medium effect size [*F*_(3,126)_ = 3.04, *P* = 0.032, η^2^_p_ = 0.067]. Post-hoc comparisons indicated that at the delayed testing session, the group that was subject to odor reinstatement (group 1) performed significantly better than the group that smelled an odor during the immediate, but not the delayed, session (group 2, *P* = 0.023) and the group that received odorless plugs at both sessions (group 4, *P* = 0.04) (see Table 1 for all descriptive statistics).

### Visuospatial episodic memory

The analysis revealed no main effect of group on performance in the “Rey-Osterrieth Complex Figure task.” The main effect of testing session was significant [*F*_(1,126)_ = 5.62, *P* = 0.02, η^2^_p_ = 0.043]. The subjects performed significantly poorer in the visuospatial memory task during immediate testing (*M* = 26.53) as compared with the picture-copying condition (*M* = 35.58) and delayed testing (*M* = 22.75) as compared with the copying and immediate testing conditions (all *P*s < 0.001) (see Table 1 for descriptive data). The interaction between group and testing session was not significant [*F*_(3,126)_ = 0.18, *P* = 0.91, η^2^_p_ = 0.004].

### Priming

We explored the effects of olfactory context cue on two types of scores in the priming task: the number of correctly completed, previously primed words (with a maximum score of 30 points) and the total number of formed words (with a maximum score of 60 points). We found no significant main or interaction effects of group and testing session on priming task performance for the correctly completed primed words [interaction: *F*_(3,126)_ = 0.69, *P* = 0.56, η^2^_p_ = 0.02] (see Table 1 for descriptive data).

### Odor pleasantness and memory scores

The results indicated no significant correlations between perceived pleasantness and performance in the three memory tasks either at immediate or at delayed testing (*P*s > 0.38). In general, participants using odorized Nosa plugs perceived the odor as rather pleasant (*M* = 5.29 ± 2.10 for the first session and *M* = 5.13 ± 2.26 for the second session), and the ratings of the odor were very similar at both testing sessions [session 1: group 1: *M* = 5.20(±2.19) vs. group 2: *M* = 5.39(±2.03), *t*_(66)_ = −0.38, *P* = 0.71; session 2: group 1: *M* = 5.09(±2.20) vs. group 3: *M* = 5.16(±2.37), *t*_(62)_ = −0.12, *P* = 0.90].

## Discussion

Our study showed that olfactory cues supported delayed recall of verbal information, and that odor reinstatement had no significant effect on visuospatial memory and priming. Different degrees of effectiveness of the odor memory cue across the included memory indices suggest that odor reinstatement can indeed affect recall processes, but also that this effect is not straightforward. It seems that verbal recall can be particularly sensitive to the enhancing effect of an odor reinstatement, but more studies are definitely needed to test how the encoding and recall of specific information interact with odorous environmental information. Nevertheless, we showed that odors do affect verbal information recall, and we suggest that it seems worthy of investigation to explore how these effects can be applied in practice.

Odor reinstatement had a slight but positive effect on verbal memory in our study. It is crucial to note here that learning of a specified verbal material is often required at school, which suggests a particular value of our results in the teaching process. An odorous cue proved to be effective also in comparison with a different, strong contextual cue (odorless nose plug) reinstated in the no odor–no odor condition. In previous studies on verbal episodic memory, a similar effect was found for recall of rosemary-related words following rosemary odor reinstatement (Stafford et al. 2009), prose recall performance when both sessions were performed on a paper impregnated with a peppermint odor (Pointer and Bond 1998), and free recall of encoded list of words in the presence of an odor (Parker et al. 2001). Interestingly, odors aided the free recall of studied words but did not increase the number of semantically related words (so-called false memories) in the Deese–Roediger–McDermott false memory paradigm (Tamminen and Mebude 2019).

We may hypothesize what drives the effects of odor cues on memory processing. Olfactory components of previous experiences can be particularly enduring, and following the “differential cue affordance value” they can serve as triggers for the entire, otherwise poorly retrieved memories (Chu and Downes 2000). They also have a particular ability to generate emotions (Hinton and Henley 1993) or affect mood (Ball et al. 2010). Autobiographical memories evoked by olfactory stimuli are characterized by a range of characteristics, summarized in the “LOVER” acronym by Larsson et al. (2014): They are typically limbic, old, vivid, emotional, and rare. Perhaps verbal information encoded and recalled in the presence of an odor may be memorized better than, for example, an abstract figure or a list of unlinked words that were to be memorized in other parts of our study because of some autobiographical olfactory memory-related effects. It may have been easier for our subjects to attribute emotional value and find some personal association between the odor they smelled and the verbal prose they encoded than to experience this connection for the abstract material in two other memory tests. Such an association was shown by Herz and Cupchik (1995), who observed that recollections of paintings encoded in the presence of an odor were more emotional than the recollections cued by words. This hypothesis finds certain support also in neurophysiological data: Personally meaningful odors evoke strong activation in the amygdala–hippocampal complex during recall (Herz et al. 2004). However, the successful encoding and retrieval of information in the presence of an odor may be also driven by increased activation in the piriform cortex during effective information encoding (Reichert et al. 2017), and it seems worthy to investigate the trends that we found in further neurophysiological research.

We want to highlight here that the effect found in our study was particularly noticeable when the group subject to odor stimulation at both sessions was compared with groups that received odorless nose plugs at retrieval. The bulk of previous work also favors the notion that the odor must be present at both encoding and recall for memory enhancement effects to occur (e.g., Eich 1978; Schab 1990; Smith et al. 1992). Nevertheless, in the verbal memory task, the performance of the group that received an odor exclusively at retrieval was merely slightly (and not significantly) poorer than that of the group smelling the odor at both sessions. The presence of an odor at retrieval could have thus affected the verbal memory performance. We may hypothesize that there are certain characteristics of the odor that we applied that can be important from the point of view of context reinstatement research (like its pleasantness or activating properties). Exposure to pleasant odors can affect mood and behaviors (for a review, see Herz 2009), and thus the effect we found could probably be partially explained by, for example, decreased anxiety and higher alertness induced by the properties of menthol. On the other hand, Ball et al. (2010) showed that performance in an indirect memory measure was enhanced by an odor cue only for an odor that was rather unpleasant and distinctive (i.e., rosemary; interestingly, the same odor was found to be an effective environmental cue by Stafford et al. [2009]). The potential odor properties that moderate the olfactory effects on memory performance should thus be explored in more detail in further research, and our findings warrant a replication with a range of different smells varying in hedonic value and arousing qualities. It could be additionally tested whether the emotional qualities of the memorized material affect the effectiveness of odors as memory cues.

As mentioned above, the implications of showing odors as effective environmental cues supporting retrieval of encoded verbal information can be particularly important in the teaching context. Odors can be helpful as cues, increasing the range of environmental information following the context-dependent processing (Tulving and Thomson 1973). Additionally, smells can not only serve as cues facilitating information recall, but also enhance the value of (concurrently presented) pictures as memory cues (Lwin et al. 2010; Lwin and Morrin 2012). Therefore, it seems worthy to explore cross-modal effects (involving cues from more than one modality) and further explore the connection between sensory perception and memory.

An indirect but crucial advantage of our research is that we present a novel, interesting method of olfactory stimulation that can be used in further memory studies. Controlled and standardized, but at the same time straightforward, presentation of odor stimuli is a problem often encountered in olfactory memory research (see Hackländer et al. 2019). The qualities of the Nosa plugs make them mostly independent of confounding environmental factors and potential differences in odor freshness or concentration across subjects during prolonged experiments. Removal of the plug or replacing it with a differently scented plug results in an immediate change of odor environment without lingering ambient odor effects; this may largely facilitate the execution of complex studies involving, for example, modifications of odor types or breaks in olfactory stimulation. It seems of utmost importance to further investigate the applicability of Nosa plugs in different types of memory studies; for example, in neurophysiological research.

We should also note certain limitations of our study. First, it needs to be mentioned that even unscented nose plugs in the control condition in our study were quite unusual for the subjects and constituted an environmental (but not an odorous) context cue. The effects of odorous contextual reinstatement may depend on the status of the olfactory context cue in relation to other cues. According to the outshining hypothesis (Smith 1988; Smith and Vela 2001), a context cue is only effective if there is a lack of other useful cues. However, if strong cues are available in the test itself (such as usage of the same task in immediate and delayed testing sessions) or in the experimental situation (like the presence of a nose plug in both the experimental and control groups in our study), olfactory contextual cues could be redundant and less effective. Therefore, it needs to be remembered that the odor reinstatement effects that we observed (or did not observe) should be interpreted also in the context of other potential memory cues. Second, our study showed a significant effect of odor reinstatement of small to medium effect size for verbal recall and nonsignificant effects for visuospatial memory and the priming task. However, this does not necessarily mean a considerable difference in the magnitude of effect sizes across the three tasks. Provided the interesting trends we observed in our data, we recommend further studies with larger samples and perhaps even more diverse memory tasks.

In summary, the present study showed that olfactory cues can be used to facilitate retrieval of declarative verbal memories. An important goal for future research is to uncover the processes that underlie this dissociation to fully understand the odor context-dependent effects on verbal information recall and to assess the value of this effect in the context of teaching.

## Conclusions

Learning processes can be affected by various factors, and here we tested whether odors affected declarative and nondeclarative memory functions assessed with three different memory tasks. We found that odor exposure aided story recall at delayed testing; that is, it was particularly valuable while recalling information typically acquired at school. Differences between the tested memory indices suggest that odor reinstatement affects specific types of recall processes. Our findings extend the knowledge on the role of olfactory cues in shaping and modulating memory and might serve as a base for further research on sensory cues aiding the learning/teaching process. An additional strength of this submission is a novel method of odor reinstatement that we applied in the study; namely, intranasal Nosa plugs.

## Materials and Methods

The study was conducted according to the guidelines of the Declaration of Helsinki on Biomedical Research Involving Human Subjects and accepted by the Ethics Committee at the Institute of Psychology, University of Wroclaw, Poland.

### Participants

The study comprised 131 participants (mean age 21.5 ± 4.22 yr; range 18–53 yr; 85.44% women) assigned randomly into one of four experimental groups. All participants were questioned about their health status and potential olfactory dysfunction as well as about their self-assessed olfactory sensitivity, and they reported no major health- or olfaction-related problems (3.47 ± 0.85 for self-assessed olfaction; range: from 1 = much poorer compared with peers to 5 = much better compared with peers). The experimental groups were found not to differ in age and self-reported smell sensitivity (*P*s = 0.47 and *P*s = 0.50, respectively). All participants were university students; they volunteered to take part in the study and provided an informed, written consent prior to the study inclusion.

### Materials

#### Olfactory context cue

As noted above, we used a novel method of olfactory stimulation; namely, Nosa plugs (https://nosaplugs.com). Nosa is a small nose plug that is typically infused with menthol odor. Here, depending on the group, the participants received either an odorized or an odorless Nosa plug. This device made the participants smell an odor while breathing through the product. All participants exposed to the menthol odor declared that they could perceive the smell while breathing.

#### Memory tests

The study comprised three different memory domains: verbal episodic memory, visuospatial episodic memory, and word fragment completion (priming). In this way, we were able to study whether potential effects of congruency at encoding and retrieval would exert differential effects on declarative and nondeclarative forms of memory and to compare whether potential context effects in declarative memory would vary as a function of the type of item information (i.e., verbal [see Pointer and Bond 1998] and visuospatial [see Reichert et al. 2017]).

##### Verbal episodic memory

The “Rivermead Behavioral Memory test” (RBMT) was used as a test of verbal episodic memory (Wilson et al. 1989; Strauss et al. 2006). The test comprises a text passage about 200 workers at a shipyard in Karlskrona that had gone on strike, and the original version possessed by the research team was in Swedish. Using the translation and back-translation procedure, the research team prepared the method in the participants’ native language—one person fluent in both Swedish and the target language translated the text, and then another person, also fluent in both languages, translated it back. Any resulting differences between the original version and the translation were discussed to find the best wording options. The final text had 58 words. During the encoding phase, the participants were instructed to read the text repeatedly for 2 min and to try to memorize it. At both the immediate and the delayed testing, participants were asked to recall the story by writing down in a few minutes as much of the prose passage as possible and to do it as accurately as they could. Following the original scoring criteria, the generated text that was divided into 21 scoring entities (Wilson et al. 2001), with a score of 21 indicating a perfect recall.

##### Visuospatial episodic memory

Episodic memory for visuospatial information was measured by the “Rey-Osterrieth Complex Figure task” (Baser and Ruff 1987; Meyers and Meyers 1999; Lezak 2004). Here, the participants were asked to closely study the presented figure and to draw a copy of this figure in a space provided below the drawing. They were given a maximum of 5 min to complete the task, and their performance in this part of the visuospatial memory task was later included as a baseline measurement in the corresponding analysis. At the immediate and the delayed recall sessions, the subjects were asked to recall and redraw the figure as accurately as possible on a blank paper. Scoring was performed both for the copy (i.e., baseline) and for both of the testing sessions, with a maximum performance of 36 points for each task (Meyers and Meyers 1999; Strauss et al. 2006).

##### Priming

In the priming task, participants were presented with a list comprising 30 words of varying length, written in capital letters (Karlsson et al. 2003). At encoding, the subjects were instructed to count the total number of As for all words presented and to put a cross after the words that were composed of six letters. The task was to be completed in 1 min. Priming (i.e., completion rate) was assessed in the testing sessions by a presentation of 60 word fragments (identical for both sessions), 30 of which originated from the encoded, original word list. The word fragments were constructed in such a way that they could be completed in more than one way. Two separate scores were obtained for the word fragment completion: the number of correctly completed primed words (maximum 30 points; score used in the further analysis) and the number of totally correct formed words (maximum 60). The participants were given 7 min to complete the task. As this test was not available in the participants’ native language, the research team performed a four-step translation of this method from Swedish (see Supplemental File S1 for a full description of this process and the list of primed words).

#### Distracter tasks

Following standard procedure in odor memory experiments (Herz and Engen 1996), we introduced a distracter task during the retention interval between the encoding and the recall sessions. In order to minimize potential effects from rehearsal on memory performance, distractor tasks were given immediately after the presentation of the information to be remembered. We used the “Digit–Symbol Substitution test” (Wechsler 1981) and the “Word Association test” (FAS) (Benton and Hamsher 1989). The “Digit–Symbol Substitution test” (Wechsler 1981) assesses attention and comprises pairs of simple symbols and digits. The task is to copy the symbols corresponding to numbers beneath a predefined series of numbers. A legend is provided to show which symbol is associated with each number. Ninety seconds were allowed to copy as many symbols as possible to designated spaces. The FAS test (Benton and Hamsher 1989) consists of three word-finding tasks, using the letters F, A, and S. The participants were instructed to write as many words beginning with each target letter as possible; they were allowed 1 min per letter.

### Design and procedure

Each participant was randomly assigned to one of four experimental conditions. The conditions differed in the provided odor cues at encoding and recall (group 1: odor at encoding–odor at recall, group 2: odor at encoding–no odor at recall, group 3: no odor at encoding–odor at recall, and group 4: no odor at encoding–no odor at recall). Memory performance for each subject was tested twice: immediately after the distracter tasks and 1 wk after encoding. At the beginning of the encoding and immediate memory testing session, the subjects received a booklet containing instructions, distracter tasks, and memory tests. They were asked to write their code at the top of the testing booklet. We guaranteed our subjects that the testing would be anonymous and that only the codes would be used to match the data from two testing sessions in our database.

All testing sessions also included a questionnaire regarding demographic information, health status, and self-rated olfactory abilities. Additionally, participants who received an odorized Nosa version rated the perceived pleasantness of its odor on a nine-point scale (1 = very repulsive to 9 = extremely pleasant) on a given testing session. The encoding and immediate testing session took ∼40 min, and the delayed recall session took ∼20 min.

The encoding and testing sessions were performed in the same room group and by the same research assistant. The order of memory tests was kept constant between participants and testing sessions.

### Data analysis

The differences in memory scores across groups were explored by means of a two-by-four mixed ANCOVA, with experimental group (1: odor–odor, 2: odor–no odor, 3: no odor–odor, 4: no odor–no odor) included as a between-subject factor, testing session (immediate vs. delayed testing) included as a within-subject factor, and self-assessed olfactory abilities included as a covariate^1^. The analyses were performed for each memory domain separately, and the analysis on visuospatial memory included an additional baseline measurement (drawing copying). To obtain a power of 0.80 with alpha level set to 0.05 to observe a medium effect of *f* = 0.25, the projected sample size was at least 128 subjects. We did not assume outlier removal. Data analyses were performed in Jamovi 2.0 (https://www.jamovi.org).

## Supplementary Material

Supplemental Material

## Footnotes

1 Following the comments we received during the peer review process, we performed all analyses also without the self-assessed smell covariate. The pattern of results was very similar to the reported findings, and detailed results of these analyses are in Supplemental File S1 (“Results Without the ‘Self-Assessed Smell’ Covariate”).
