# Supplementary material for: Odor-based context-dependent memory: influence of olfactory cues on declarative and nondeclarative memory indices
Source: Learn Mem. 2022 May;29(5):136–41. doi: 10.1101/lm.053562.121 (PMC9053110; doi:10.1101/lm.053562.121)
Supplement: Supplemental Material [file supp_29_5_136__DC1.html]

Supplemental Material 

# Odor-based context-dependent memory: influence of olfactory cues on declarative and nondeclarative memory indices

## Supplemental Material

- Supplemental\_File.docx
