# Supplementary material for: Odor-based context-dependent memory: influence of olfactory cues on declarative and nondeclarative memory indices
Source: Learn Mem. 2022 May;29(5):136–41. doi: 10.1101/lm.053562.121 (PMC9053110; doi:10.1101/lm.053562.121)
Supplement: Supplemental Material [file supp_29.5.136_Supplemental_File.docx]

**Supplementary File 1**

1. **Priming task**
2. ***Priming task translation procedure***

First, two native Swedish speakers assessed the commonness of each word used in the original task in Swedish language. Second, the words were translated to the participants’ language using the translation and back-translation procedure. Third, a panel of six native speakers of the target language prepared a final version of the scale, based the translated Swedish originals. Each new word had to: (a) have the same number of letters as the original word, (b) have the same meaning / be semantically close or similar to the original word, (c) be equally common in Polish and in Swedish. Whenever it was possible, the same words (e.g., banan in Swedish and banan in Polish) or their direct translations were used (e.g., makrill in Swedish and makrela in Polish). However, sometimes the words had to be replaced (e.g., Swedish word “svart”, meaning “black” was too long in Polish (“czarny”), therefore it was replaced with “white”, i.e., “biały” – a word equally long and common as the Swedish original and semantically close to this word. Finally, mirroring the original procedure, several letters were removed from each word, so that each test item could be completed in at least two different ways.

1. ***List of words***

The list of all applied words translated to English is presented below:

Peony

Sweet

White

Banana

Fat

Latch

Triangular

Table

Little doll

Camel

Nice

Coffee maker

Distant

Tasty

Grill

Duck

Bright

Violet

Beetroot

Awful

Mackerel

Trouble

Strong

Lamp

Crocodile

Idler

Drawer

Couch

Large

Spade

1. **Results without the “self-assessed smell” covariate**

*3.1.1. Verbal episodic memory*

There was no main effect of group on the performance in the RBMT episodic memory test, but we found a significant main effect of the testing session, *F*(1,127)=114.38, *p*<.001, η^2^_p_=.47, with participants in all groups recalling significantly more information during the immediate testing session. We also observed a marginally significant interaction between group and testing session, *F*(3,127)=2.60, *p*=.055, η^2^_p_=.058, with post-hoc comparisons indicating that at the delayed testing session the group that was subject to odor reinstatement (Group 1) performed significantly better than the group that smelled an odor at encoding, but not at the delayed session (Group 2), *p*=.023, and the group that received an odorless plug at both sessions (Group 4), *p*=.04.

*3.1.2. Visuospatial episodic memory*

The analysis revealed no main effect of group on performance in *the Rey-Osterrieth Complex Figure task*. The main effect of testing session was significant, *F*(1,127)=122.40, *p*<.001, η^2^=.49. Neither the group nor the testing session*group effect were significant.

*3.1.3. Priming*

We found no significant main or interaction effects of group and testing session on priming task performance for the correctly completed primed words.

1. **Analysis with “odor at encoding” and “odor at retrieval” factors**

There were no significant main or interaction effects of “odor at encoding” and “odor at retrieval” on the RBMT episodic memory test during the immediate recall session, as well as on performance in the Rey-Osterrieth Complex Figure task and the priming task neither at the immediate nor at delayed testing sessions. We found a significant main effect of “odor at retrieval” for RBMT episodic memory test at the delated testing session, *F*(1,127)=6.71, *p*=.011, with participants recalling significantly more information in odor presence. Consistent with the analysis presented in the main manuscript file, post-hoc comparisons indicated that this effect was driven by a significant advantage of the group that was subject to odor reinstatement (Group 1, odor-odor) over the group that smelled the odor at encoding, but not at the delayed session (Group 2, no odor-odor), *p*=.023, and over the group that received an odorless plug at both sessions (Group 4), *p*=.04.
